# COVID-19 Vaccination and Ukrainian Refugees in Poland during Russian–Ukrainian War—Narrative Review

**DOI:** 10.3390/vaccines10060955

**Published:** 2022-06-16

**Authors:** Wojciech Malchrzak, Mateusz Babicki, Dagmara Pokorna-Kałwak, Zbigniew Doniec, Agnieszka Mastalerz-Migas

**Affiliations:** 1 Department of Family Medicine, Wroclaw Medical University, Syrokomli 1, 51-141 Wroclaw, Poland; wojciech.malchrzak@gmail.com (W.M.); daga_kalwak@o2.pl (D.P.-K.); agnieszka.migas@gmail.com (A.M.-M.); 2 Department of Pediatric Pneumonology and Allergology, Institute of Tuberculosis and Lung Diseases Regional Branch, 34-700 Rabka Zdroj, Poland; zdoniec1@gmail.com; 3 Medical Institute, Podhale State College of Applied Sciences, 34-400 Nowy Targ, Poland

**Keywords:** COVID-19, COVID-19 vaccination, refugees

## Abstract

The outbreak of the Russian–Ukrainian war contributed to the largest migration movement in the 21st century. As a result, over 3 million refugees, mainly women, children and the elderly, arrived in Poland in a short space of time. Despite the ongoing war, it is important to remember that the COVID-19 pandemic is still present in the world, and before the outbreak of the war, Ukraine was struggling with its fifth wave. Furthermore, Ukraine has one of the lowest vaccination rates in Europe, not exceeding 40%. It is, therefore, reasonable to suspect that the vast majority of migrants have not been vaccinated. This situation may pose a significant epidemiological risk. Therefore, it is necessary to implement appropriate steps to determine the vaccination status of refugees and to supplement the vaccination with both the core and booster doses. In response to these needs, the government of Poland, like many other countries, has made it possible to provide free COVID-19 vaccination to persons fleeing war. In the face of massive migration, the overriding priority should be to ensure adequate medical care for refugees, including free COVID-19 vaccinations. However, it seems that the lack of willingness to vaccinate among Ukrainians is also replicated on migration. It seems reasonable that appropriate steps should be taken to increase awareness and confidence in vaccination, which may ultimately translate into increased vaccination uptake. Analyzing previous experiences, it is advisable to consider that the first step should be to promote vaccination and remind refugees of the possibility of free COVID-19 vaccination. Additionally, refugees should be encouraged to be vaccinated during every contact with health care workers.

## 1. Introduction

Due to the ongoing armed conflict in Ukraine since February 2022, more than three million refugees have arrived in Poland in 3 months [1]. This is the largest migration movement in the history of Europe. This situation brings many potential risks, including epidemiological ones. Firstly, the period of the outbreak coincided with the peak of the 5th wave of the pandemic in Ukraine and with daily incidence rates of 30–40 thousand [2]. Secondly, the failure of the COVID-19 vaccination programme, with a full vaccination rate of less than 40% of the population [3], presents another risk. Thirdly, hundreds of thousands of people trying to leave the country waited at the border, where it was impossible to maintain a sanitary regime or social distance. This created ideal conditions for the spread of SARS-CoV-2.

Moreover, during this period, Poland still required a negative PCR or antigen test for people arriving from outside the Schengen zone before crossing the border [4]. However, the Polish government abolished the quarantine requirement for refugees, and they were exempted from testing for SARS-CoV-2 infection before crossing the border. Showing proof of vaccination was also not mandatory [5]. This led to the situation that, in a short period of time, 3 million people with unknown vaccination status arrived in Poland without any sanitary control.

Based on the COVID-19 vaccination data in Ukraine, it should be suspected that the vast majority of the refugees did not receive the vaccination [2]. Therefore, it is necessary to implement appropriate steps to determine the vaccination status of refugees and to supplement the vaccination with both the core schedule and the booster dose.

To the best of the authors’ knowledge, no scientific summary of the impact of refugee migration on the COVID-19 outbreak in Poland has been published to date. At the same time, the Polish Government does not maintain a register with information on the vaccination status of persons crossing the border, hence the lack of objective sources allowing for a direct assessment of the potential threat to public health. The purpose of this publication is to summarize the available information and to provide a synthesis of this issue. Poland has not established a system to manage public health risks associated with increased migration. An example of such a system is the one established in Italy during the migration crisis in 2010. The use of a syndromic surveillance system confirmed its effectiveness in protecting public health [6].

## 2. Methodology

This study is an unstructured literature review that used as its source materials not only scholarly publications, but also statistical data from public institutions, press materials, including publications in the social media, and legal regulations, as well as content from official government websites in both Poland and Ukraine.

## 3. Organization of Vaccination against COVID-19 in Ukraine

The vaccination programme against COVID-19 in Ukraine started on 24.02.2021 [7]. Six preparations were approved for vaccination: mRNA-based Comirnaty (Pfizer) and Spikevax (Moderna), COVID-19 adenoviral vector-containing Vaccine Janssen, Vaxzevria (AstraZeneca) and its counterpart Covishield, as well as inactivated vaccine CoronaVac (Sinovac) [8]. These preparations were authorised and used following the summary of product characteristics.

According to the objectives of the vaccination programme, the priority group was comprised of medics working in hospitals and military personnel. Then, vaccination was allowed to people from the oldest age groups, i.e., over 80 years old. With time, younger and younger people were allowed to be vaccinated, and finally, after 2–3 months, the vaccinations were made available to other medical groups. According to the plan, in November–December 2021, vaccination would be available to all eligible persons [9]. In reality, the implementation of the programme was delayed, mainly due to the low availability of the preparations [10]. However, this improved over time and, finally, in July 2021, it was announced that vaccination would be available to all those willing over 18 years of age [11,12].

Furthermore, in October 2021, adolescents aged 12–17 years could also benefit from vaccination (Comirnaty was authorised) [13]. Moreover, in 2021/2022, a booster dose was approved for persons ≥18 years of age who have been at least 6 months past the date of the previous vaccination [14]. Despite the available formulation, vaccinations for children aged 5–11 years have not been initiated in Ukraine [7].

## 4. Attitudes toward Vaccination against COVID-19 in Ukraine

In research conducted at the very beginning of the universal vaccination program, i.e., in January 2021, the willingness to be vaccinated against COVID-19 was declared by 53–81% of citizens, and the most preferred vaccines were, respectively, Vaxzevria and its counterpart Covishield (AstraZeneca), Spikevax (Moderna), CoronaVac (Sinovac), Comirnaty (Pfizer) and, finally, the vaccine of the Gamely company Sputnik V, which was finally not registered in Ukraine [15]. In a survey conducted two months later, the percentage of those willing to be vaccinated was 63%, of which 31% only with a specific preparation [16]. Despite higher initial projections, in November 2021, after more than eight months of universal vaccination, 20% of the population had been vaccinated. Survey results published at the time showed that 40–60% of the population did not intend to be vaccinated against COVID-19 [17]. Several explanations were postulated, one of which was supposed to be a Russian disinformation campaign, which, to weaken Ukraine, was supposed to discourage people from getting vaccinated and thus expose more Ukrainian citizens to the infection. It is also noteworthy that the activities of antivaccination movements in Ukraine have been evident since the turn of the 20th century. The result of this may be, among others, a 53% vaccination rate against poliomyelitis [18].

The lack of confidence of Ukrainians in vaccination cannot be overlooked either. Fears of adverse reactions were reported by 44% of respondents, and more than half believe that COVID-19 vaccines have not been sufficiently tested [17]. In addition, the Ukrainian authorities do not have much trust among citizens after a series of incidents between 2010 and 2013 when, through improper storage and administration of vaccines, many people developed adverse reactions, and the authorities tried to silence the situation [17]. There is no doubt that citizens’ lack of trust in both the vaccines themselves and in those responsible for vaccination organisations is the primary reason for reluctance to vaccinate, regardless of the underlying sources of these concerns [19].

At the same time, the family doctor turned out to be a valuable source of knowledge for many respondents. As many as 65% indicated that they trust their family doctor, and in the group of people over 60 years of age, the percentage was 72% [16]. All this translated into the failure of the vaccination programme in Ukraine. At the outbreak of the war, only 37% of citizens were vaccinated against COVID-19, and the booster dose was received by 1.7% of those eligible [18]. Large numbers of unvaccinated individuals may transmit the virus more easily than vaccinated individuals, and given that some SARS-CoV-2 infections are asymptomatic, they may unknowingly infect more people [20]. In the face of the migration crisis, this also translates into a higher chance of outbreaks among refugees, but also an increase in the number of cases among citizens of the countries to which the refugees have arrived. Therefore, one of the public health priorities should be to make COVID-19 vaccination available to refugees in every country where they find refuge for the duration of the war.

## 5. The State of Vaccination against COVID-19 in Poland and the Possibility of Vaccination for Refugees

At the onset of the war in Ukraine, in Poland, the full COVID-19 vaccination schedule was completed by 59% of the population, and a booster dose was received by 30% of the population [18]. This result is not only lower than the European average, but one of the worst in Europe, including the central-eastern part. Higher vaccination rates have been achieved in the Czech Republic, Lithuania, and Belarus [21].

In Poland, five vaccines against COVID-19 are approved for use, the mRNA-based Comirnaty (Pfizer) and Spikevax (Moderna), the adenoviral Vaxzevria (AstraZeneca) and COVID-19 Vaccine Janssen and the latest approved subunit vaccine Nuvaxovid by Novavax [22].

The COVID-19 vaccination programme is free and available to all adults, as well as adolescents and children aged 5 years and over [23,24]. Adults can benefit from all available vaccines. Except for Janssen’s single-dose COVID-19 vaccine, the schedule includes the administration of two doses of vaccine and, in immunocompromised individuals, the third dose as part of the core schedule. At least six months after completion of the core vaccination (or two months after receiving COVID-19 Vaccine Janssen), a booster dose may be given [25]. Adolescents aged 12–17 years can benefit from Comirnata and Spikevax vaccines similarly to adults. Children aged 5–12 years are vaccinated with the paediatric version of Comirnaty, also in a two-dose schedule [24].

In the early stages of the COVID-19 vaccination programme, there was huge interest. Hundreds of thousands of doses were administered each day. However, as the programme progressed, vaccine uptake declined, and is currently approximately 7000 doses per day [26].

Since the beginning of the pandemic, over six million SARS-CoV-2 infections have been confirmed in Poland, of which over 116,000 have been fatal. Currently (June 2022), dozens of cases per day are being detected, with poje-dyne deaths from COVID-19 [27]. Importantly, the reporting system was also changed from daily to weekly. It should be noted, however, that extensive testing for SARS-CoV-2 infection has been discontinued in Poland since March 2022. Currently, PCR testing can be performed free of charge prior to hospital admission, but the decision to perform the test rests with the hospital itself. In outpatient clinics, only antigen tests can be performed, but since the withdrawal of the obligation to isolate or quarantine, their importance has diminished significantly. All of this raises serious doubts about the reliability and validity of the reported cases of both COVID-19 infections and reconciliations.

In response to the ongoing migratory crisis, the Polish government introduced a regulation allowing the administration of free COVID-19 vaccination to refugees. Due to the single-dose schedule, the primary product perforated by the government was COVID-19 Vaccine Janssen. However, depending on individual preference, vaccination with any product permitted in the European Union is possible [28]. Unfortunately, according to initial data, when the number of refugees in Poland reached two million, 2600 vaccinations against COVID-19 were performed [29]. In April, with 2.7 million refugees in Poland, the number of vaccinations among them rose to 35,400, with more than 2000 among children and adolescents [30]. These data indicate that people who have not yet been vaccinated in Ukraine are probably still not planning to do so in Poland.

In addition to vaccination against COVID-19, refugees from Ukraine were allowed to benefit from the entire health care system, including free vaccinations included in the Polish vaccination calendar. According to the law, these vaccinations are mandatory when staying in Poland for at least three months, but willing individuals can already benefit from these vaccinations earlier [31,32].

Concerning the migration crisis, the World Health Organization has announced key public health aspects to focus on. These are the continuation of vaccination programmes, the inclusion of refugees in local vaccination programmes, a special focus on vaccination against poliomyelitis, measles and rubella, and the preparation of accessible and understandable tools outlining the benefits of vaccination to encourage as many refugees as possible to be vaccinated [33].

The current situation with refugees is reminiscent of one of the earlier migration crises—the one that occurred in Europe due to the events in Syria. The large influx of people had a significant impact on public health in European countries. Each country coped differently, but the most recurrent challenges were a shortage of health system resources, a lack of adequate funding, and the need to manage multiple and diverse health problems simultaneously. Not all health systems were prepared for this public health challenge [6,34]. Similarly, during the current crisis, the Polish government has taken some steps to address the health needs of refugees, but these may not be sufficient in the long term. Refugees in Poland have access to free services, but they are not actively encouraged to attend to preventive care [35,36]. In the context of COVID-19, this means the possibility of free vaccination, but refugees are not informed or encouraged about this in any meaningful way.

The consequences of such a large number of people not vaccinated against COVID-19 combined with the low uptake of vaccination in Poland may lead to a number of consequences. First and foremost, a lower proportion of the population vaccinated means a higher risk of further local or, in extreme cases, national outbreaks. Unsatisfactory vaccination rates among refugees may also have an adverse effect on Poles who have considered vaccination. Without seeing the significant popularity of vaccination among refugees or information campaigns, they will not be motivated to vaccinate. Another effect could be the mutation of the virus, which can replicate unhindered in an unvaccinated population, and possibly the emergence of new, more dangerous variants of SARS-CoV-2.

As recommended by the World Health Organization, countries that have agreed to accept refugees provide them with vaccination against COVID-19. As in Poland, in many European countries Ukrainian citizens can be vaccinated against COVID-19 free of charge with vaccines available in the country. The exception is the Czech Republic, where the vaccination against COVID-19 for foreigners from November 2021 onwards is payable, about EUR 15 depending on the city [37,38].

The authors are aware of the limitations of this review, which is undoubtedly the narrowing of the description to an evaluation of COVID-19 vaccination only. Clearly, a comprehensive review of attitudes and needs toward immunization among refugees is necessary.

In conclusion, the armed conflict, coupled with the ongoing COVID-19 pandemic, is a huge challenge for all countries, especially those affected by the migratory crisis. In the face of massive migration, the top priority should be to provide adequate medical care for refugees, including free vaccination against COVID-19. However, it seems that the lack of willingness to vaccinate among Ukrainians is also replicated on migration. This is indicated by the very low number of vaccinations performed among refugees. It seems reasonable to take appropriate steps to increase awareness and confidence in vaccination among refugees, which may ultimately translate into increased vaccination uptake. Analyzing previous experiences, it would be advisable to consider an educational campaign promoting vaccination, as well as reminding refugees of the possibility of free vaccination against COVID-19. Additionally, refugees should be encouraged to vaccinate during every contact with health care workers [39,40,41]. Constant monitoring of the phenomenon of attitudes towards vaccination, not only against COVID-19, is very important. On the one hand, it allows to know the needs of the population in this regard and to react immediately and appropriately. On the other hand, having the preparation is not equivalent to its use, which requires social acceptance [42].

## 6. Conclusions

The war in Ukraine and the ensuing migration crisis pose a major public health challenge in Poland. Low vaccination rates against COVID-19 in Ukraine and only slightly better in Poland reduce the ability to effectively control the epidemic. Supplementary COVID-19 vaccination in the near future should be a priority for the health systems of countries affected by the refugee crisis, and according to WHO recommendations, this vaccination should be free and widely available. The efforts put into promoting vaccination among refugees, which basically stopped at allowing them to be vaccinated for free, seem to be insufficient to convince the majority of refugees to be vaccinated.

## Data Availability

Not applicable.
